# Comment on Hosmann et al. 5-ALA Fluorescence Is a Powerful Prognostic Marker during Surgery of Low-Grade Gliomas (WHO Grade II)—Experience at Two Specialized Centers. *Cancers* 2021, *13*, 2540

**DOI:** 10.3390/cancers13225634

**Published:** 2021-11-11

**Authors:** Walter Stummer, Christian Thomas

**Affiliations:** 1 Department of Neurosurgery, University of Münster, 48149 Münster, Germany; 2 Institute of Neuropathology, University of Münster, 48149 Münster, Germany; christian.thomas@ukmuenster.de

With great interest, we have read the paper by Hosmann et al. recently published in Cancers [1]. 

The authors retrospectively assess progression-free survival, time to malignant transformation and overall survival in 59 patients with histologically confirmed low-grade gliomas (LGG) operated on using 5-aminolevulinic acid (5-ALA) for fluorescence-guided resections. While the general expectation is that 5-ALA induced porphyrin fluorescence is linked to malignancy and that LGG do not normally show fluorescence, Hosmann et al. report a subcohort of histologically verified low-grade gliomas that demonstrate variable degrees of fluorescence during surgery. Importantly, prognosis regarding time to progression, malignant transformation and ultimately death were markedly worse in patients with fluorescing as opposed to non-fluorescing LGG. 

This is an important contribution to the field. However, we believe that the authors underestimate the value of their observation for hypothesis generation regarding prognosis and possibly the necessity of adjuvant cytotoxic therapy after surgery in this subgroup of patients.

They state that their study is “the first study investigating the value of 5-ALA fluorescence in pure LGG in two specialized independent centers with a sufficiently long follow-up time for newly diagnosed LGG”. However, they appear to have overlooked the fact that we published a comparable study [2] with very similar results in 2018 in *Neurosurgery*, with a completely independent, larger cohort (*n* = 74) of patients with histologically verified low-grade gliomas diagnosed according to the 2016 classification of brain tumors. Sixteen of these LGG showed fluorescence compared to seven in Hosmann’s cohort.

Importantly, our observation of inferior outcome regarding progression-free survival, time to malignant transformation and overall survival in patients with fluorescent low-grade gliomas was well mirrored in Hosmann et al.’s subsequent, independent investigation (Table 1). This markedly strengthens our initial observations and conclusions.

The one difference, however, is our larger subgroup of IDH wildtype diffuse astrocytomas. Hosmann et al. suggest a relationship between IDH mutation status and fluorescence. By contrast, we adjusted the influence of IDH mutation status by a variety of covariates and failed to find such a relationship. In our study, IDH mutational status and fluorescence were both independent prognostic factors for survival. This observation is noteworthy. Diffuse astrocytomas, be they IDH mutated or wildtype, tend to develop malignant transformation over time. IDH mutational status is normally considered a stable molecular change throughout progression and malignant transformation of a glioma [3], whereas fluorescence appears to be a marker of incipient progression and malignant transformation and thus appears to provide a temporal indicator regarding a tumor’s near future.

The large number of IDH-mutated diffuse astrocytomas in Hosmann’s cohort, which have a better prognosis per se [4], also helps explain the apparently better outcomes in non-fluorescing diffuse astrocytomas in their study.

Together, from our data, which Hosmann et al. now corroborate in an independent cohort, we hypothesize that fluorescence heralds malignant transformation and worse prognosis in patients with diffuse low-grade gliomas, independently of IDH mutation status. The molecular mechanisms behind this phenomenon are unclear. In our earlier investigation, we failed to find a convincing association with commonly determined molecular markers for gliomas, such as the MIB index, 1p19q co-deletions, p53 mutations, ATRX loss and EGFR expression [2].

It is common practice to treat patients with IDH-mutated diffuse astrocytomas with subsequent radio- and chemo-therapy, based on data from the RTOG 9802 study [5], when patients are >40 years of age and/or have residual tumor after surgery (high-risk patients). We believe that this simple stratification may lead to the overtreatment of some patients and undertreatment of others. We hypothesize that 5-ALA-induced tumor fluorescence should be further explored as an independent indicator of high-risk patients with LGG, and in such patients, adjuvant radio- and chemo-therapy should be considered as part of their treatment regime.

## Figures and Tables

**Table 1:** Comparison between outcome data regarding fluorescing and non-fluorescing low-grade glioma after surgery from Jaber et al. [2] and Hosmann al. [1]. Note higher fraction of IDH-mutated tumors in Hosmann’s cohort.

	Jaber et al. [2]			Hosmann et al. [1]		
	Flu+	Flu−	*p*	Flu+	Flu−	*p*
N	16	58		7	52	
*n* (%) IDH mutated	9 (56.3)	37 (63.8)	0.86	5(71)	49 (94.2)	0.04
Observation period (months)	46.4 [41.8–51.1] *			63.6 ± 34.8 **		
Time to malignant transformation (months)	43.0 [27.5–58.5] **	64.6 [57.7–71.5] **	0.02	46.8 ± 8.4	96 ± 7.2 **	0.03
Overall survival (months)	51.6 [34.8–68.3]	68.2 [62.7–73.8]	0.01	64.8 ± 20.4	123.6 ± 6.0 **	0.01

* medians [95% confidence interval], ** means [95% confidence interval] (conversion from years to months by the authors).
